# Are multiple views superior to a single view when teaching hip surgery? A single-blinded randomized controlled trial of technical skill acquisition

**DOI:** 10.1371/journal.pone.0209904

**Published:** 2019-01-09

**Authors:** Huixiang Wang, Kapil Sugand, Simon Newman, Gareth Jones, Justin Cobb, Edouard Auvinet

**Affiliations:** 1 MSK Lab, Imperial College London, Charing Cross Hospital, London, United Kingdom; 2 Orthopaedic Traumatology, Trauma Center, Shanghai General Hospital, School of Medicine, Shanghai Jiao Tong University, Shanghai, PR China; Medical University Graz, AUSTRIA

## Abstract

**Purpose:**

Surgical education videos currently all use a single point of view (POV) with the trainee locked onto a fixed viewpoint, which may not deliver sufficient information for complex procedures. We developed a novel multiple POV video system and evaluated its training outcome compared with traditional single POV.

**Methods:**

We filmed a hip resurfacing procedure performed by an expert attending using 8 cameras in theatre. 30 medical students were randomly and equally allocated to learn the procedure using the multiple POV (experiment group [EG]) versus single POV system (control group [CG]). Participants advanced a pin into the femoral head as demonstrated in the video. We measured the drilling trajectories and compared it with pre-operative plan to evaluate distance of the pin insertion and angular deviations. Two orthopedic attendings expertly evaluated the participants’ performance using a modified global rating scale (GRS). There was a pre-video knowledge test that was repeated post-simulation alongside a Likert-scale questionnaire.

**Results:**

The angular deviation of the pin in EG was significantly less by 29% compared to CG (p = 0.037), with no significant difference in the entry point’s distance between groups (p = 0.204). The GRS scores for EG were 3.5% higher than CG (p = 0.046). There was a 32% higher overall knowledge test score (p<0.001) and 21% improved Likert-scale questionnaire score (p = 0.002) after video-learning in EG than CG, albeit no significant difference in the knowledge test score before video-learning (p = 0.721).

**Conclusion:**

The novel multiple POV provided significant objective and subjective advantages over single POV for acquisition of technical skills in hip surgery.

## Introduction

Surgical education has traditionally been on an apprenticeship model in which residents are given increasing autonomy while performing live surgical cases at the discretion of a supervising surgeon [1, 2], but in the past decades there has been a dramatic reconfiguration of surgical training driven by (i) increased public awareness of iatrogenic errors and lawsuits [3], (ii) restriction of duty hours due to the European Working Time Directive (EWTD) and American Accreditation Council for Graduate Medical Education, with an estimated 80% decrease in dedicated operating time as a result of EWTD [4, 5], (iii) an emphasis on efficient use of theatre time due to cost pressures [6,7]. Additionally, the visible surgical field is practically often limited, and it is difficult to have an adequate view to facilitate training [8]. Consequently, developing surgical training methods away from the operating theatre is being considered.

Outside operating theatres, orthopedic trainees practice technical skills on cadavers and sawbones. However, these teaching techniques are limited by the expense and short supply of cadavers, and the lack of high-fidelity of sawbones. Recently, virtual reality (VR) simulators have provided trainees with the opportunity to practice and overcome the learning curve in a high-fidelity, safe and controlled environment without compromising patient safety [9–13]. But the expense associated with their purchase and maintenance and lack of realism has restricted their use. Also, in orthopedic specialty, VR simulators are mostly suitable for teaching basic psychomotor skills in arthroscopy and fracture fixations [14–18], whilst the majority of orthopedic interventions consist of open procedures (such as arthroplasty) with complex anatomy that are not easily amenable to simulation yet. Consequently, adjunctive methods of acquiring surgical skills away from the operating room are necessary without compromising patient safety.

One solution to supplement surgical training has been the use of videos which have become increasingly popular because of their low cost and easy access. Existing video systems all use a single point of view (POV) with 2D or 3D rendering [19–23] whereby the trainees are locked into a single viewpoint. However, some open procedures, such as arthroplasty, require visuospatial awareness in multiple dimensions and from multiple viewpoints. Single and fixed POV may therefore not be sufficient to provide the necessary information for training purposes.

The challenge in surgery teaching resides in teaching both knowledge and spatial perception and gesture transmission. There by, to assess a 3D information such the orientation of a tool in space versus anatomical references, single POV video might reduce the exposure of the student to essential information.

The objective was to develop a novel multiple POV video system and to evaluate the training outcome of multiple versus traditional single POV. The following three outcomes were observed:

- Knowledge acquisition of the operative steps with a questionnaire
- Real-time technical and objective performance metrics achieved after completing the learning session with a GRS score marked by two expert orthopaedic surgeons
- Assessing the precision of pin positioning

The null hypothesis was observing no difference in technical skill and knowledge acquisition between single (control group) and multiple POV (experimental group) videos.

## Materials and methods

### Video recordings and development of a multiple POV video system

The following two procedures were recorded:

- Recording of a procedure performed on a sawbone by an expert orthopedic surgeon with clear explanations of each step,
- Recording of an actual hip resurfacing procedure in theatre, performed by an expert orthopedic surgeon (>20 years of experience).

Both videos were recorded by 14 commercial cameras (12 JVC HD camera, Yokohama, Japan; 2 Go Pro Hero 4 Black, California, USA) arrayed around the operating table after getting informed consent from the patient for the second video. Of these, 12 were fixed to the plastic laminar flow hood which surrounds the operating table, with one fixed to the handle of the surgical lamp and the remaining one mounted to the surgeon’s head. After review, 8 cameras considered to have captured optimal viewpoints were retained, whilst the remaining 6 were excluded. Based on the videos recorded from 8 POVs, a novel multiple-view video system was developed, which allowed the subject to switch between viewpoints. For mono-video control group, the view point was the camera attached to the light pole. No identifiable information or image of the patient was retained in the video. The individuals in this manuscript have given written informed consent (as outlined in PLOS consent form) to publish these case details.

### Participant recruitment and randomization

**Fig 1:**
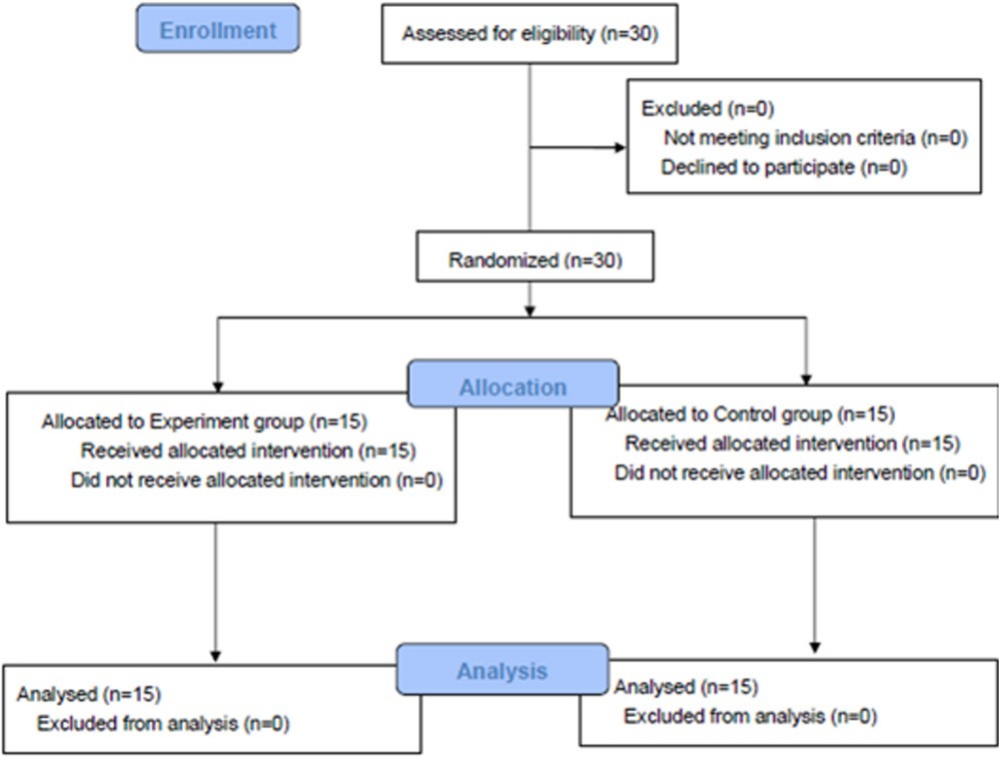
Recruitment process. Schema of the recruitment process.

The recruitment followed the process described in Fig 1. 30 student doctors were voluntarily recruited from Imperial College London. The designated tasks included learning the procedure using either multiple versus single POV video before conducting the guide wire insertion part of the operation on a simulated sawbone model. All participants were blinded with respect to the nature of the two arms of the study. Participants were randomly allocated to two different groups: (i) experiment group (multiple POV) and (ii) control group (single POV). They were given a unique identification number and randomly allocated to each cohort using a random number generator (Excel, Microsoft, Washington state, USA) by the research coordinator.

#### Date of study

January to March 2016.

#### Ethical approval and consent

The study was approved by the Medical Education Ethics Committee at Imperial College and given the reference number MEEC1415-32. Written informed consent from every participant was obtained, in which they agreed on the recording of their procedures and the use of the footage for research purposes only. The individual in this manuscript has given written informed consent (as outlined in PLOS consent form) to publish these case details.

#### Enrollment criteria

Inclusion criteria included undergraduate year 3–6 student doctors naive to both hip resurfacing and related simulations.

Exclusion criteria included previous exposure to hip resurfacing or related simulations.

### Evaluating the efficacy of the video systems

**Fig 2:**
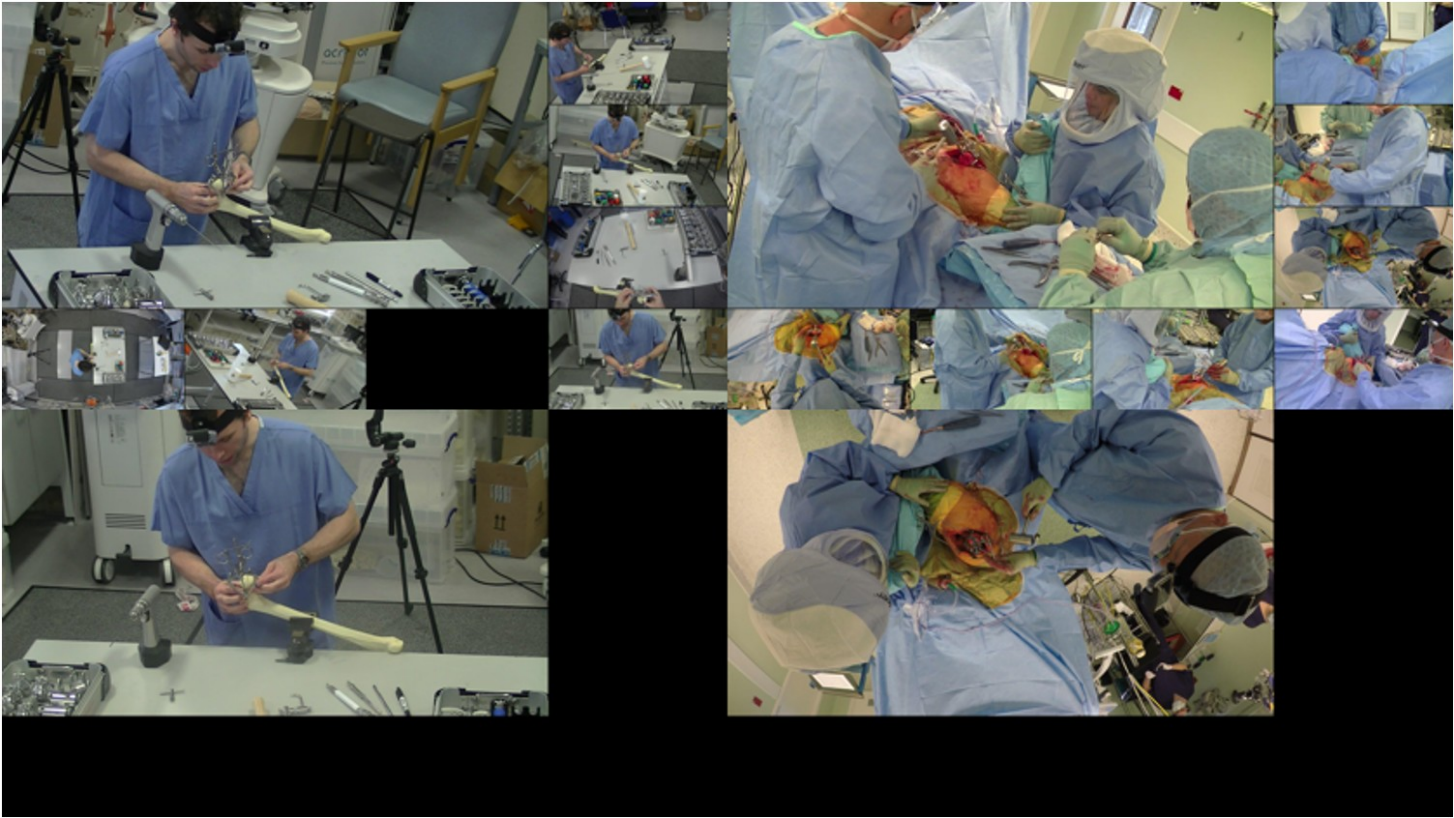
View of the single and the multiple view interface. Interface of the novel multiple POV video system, which allows the user to click and navigate through any desired viewpoint, and the equivalent for standard single POV video system.

Participants initially completed a multiple-choice test (S1 File) which tested their baseline knowledge of a hip resurfacing procedure. The test was designed using a modified Delphi technique with advice and feedback sought from four expert orthopedic attending surgeons. The experimental group (respectively control group) were asked to carry out the following tasks:

1. View a multiple POV (resp. single POV) video on hip resurfacing procedure composed with a tutorial video captured with several cameras (resp. a front camera) for the simulated sawbone and with a real hip resurfacing procedure as captured a group of cameras (resp. by the camera mounted to the handle of the surgical lamp) (as show on Fig 2). They were allowed to rewind and fast-forward the video as they wish. The viewing time was twenty minutes.
2. Complete the same multiple-choice test again, followed by a Likert-scale subjective questionnaire (S2 File) designed using a modified Delphi technique to demonstrate self-perception of their confidence and satisfaction of the learning experience.
3. Repeat the procedure on a sawbone by inserting a pin into the femoral head as demonstrated in the video, with their performance recorded by a camera (with their face or identical information not included in the video).
4. Watch the video seen by the opposite group before answering a subjective questionnaire to compare their preferences.

The sawbone models were then CT scanned to evaluate the drilling accuracy within the femoral head. The CT data (in Digital Imaging and Communications in Medicine [DICOM] format) was obtained and imported into Mimics (Materialise NV, Leuven, Belgium) software for 3D reconstruction. On each 3D model the actual drilling trajectories were extracted and compared to an ideal virtual drilling path set by the operating surgeon in the video. The difference in the distance (mm) of points of pin insertion into the femoral head, and the axis angle deviation between the actual drilled trajectory and the ideal virtual drilling path were then measured.

Both single and multiple video systems included identical verbal commentary about the surgical procedure, including how to achieve an ideal drilling trajectory in the femoral head as demonstrated by the expert surgeon.

2 expert surgeons experienced in hip resurfacing surgery evaluated the participants’ performance using the modified global rating scale (GRS) (S3 File), which was designed using a modified Delphi technique, after watching their performance recorded in the video.

### Outcomes

Primary outcome was the objective performance metrics of the deviation between participants’ trajectory and ideal virtual trajectory of pin insertion into the femoral head. Secondary outcomes were (i) the GRS scores, (ii) knowledge test scores and (iii) questionnaire scores.

### Power study

Based on a previous pilot study (n = 5) modelled on non-inferiority/superiority trials, a minimum number of 9 participants were required in each cohort (mean scores of 7.2 [experimental group] vs. 5.5 [control group], SD 1.27, 1-β = 80%, α = 5%). In the absence of a figure quoted in current literature, our pilot study suggested that a difference of 30% in the objective test scoring can be construed as being clinically significant, which was agreed by 3 expert orthopedic surgeons.

### Statistical analysis

All data were analyzed using SPSS Statistics version 19 (IBM, New York, USA). For normally distributed data an independent t-test for dependent variables was adopted, while for data that were not normally distributed, a Mann-Whitney U test was used. Results for testing were calculated as mean (SD) [95% CI] and as median ± IQR (95% Bonett Price CI) for the Likert-scale questionnaire. Results are also displayed using box-and-whisker plots and bar charts. Statistical significance was set as p<0.05.

### Dataset

The dataset of this study is available (S4 File).

## Results

### Demographics

30 participants were successfully randomized and completed the experiment. The median age for the participants was 22 (range 19–24) years and their median undergraduate year in medical school was year 4 (range 3–5). Subgroup analysis showed that 47% (n = 14) participants were male and 93% (n = 28) of them were right-handed.

### Outcomes

#### Primary outcome results

The differences in the distance between points of pin insertion into the femoral head, and the axis angle deviation between the actual drilled trajectory and the ideal virtual drilling path in both groups are shown in Table 1.

**Table 1:** The angular deviation and distance deviation of entry points between the actually drilled and ideal virtual trajectory of the pin insertion in the femoral head in both groups.

Primary outcomes	Control group Mean (SD) [95% CI]	Experiment group Mean (SD) [95% CI]	Percentage (%) improvement	p-value
**Angular deviation (degree)**	9.0 (3.1) [7.5–10.5]	6.4 (3.5) [4.6–8.1]	29	0.037
**Distance deviation (mm)**	6.8 (2.7) [5.4–8.2]	5.5 (2.6) [4.2–6.8]	18	0.204

#### Secondary outcome

**Fig 3:**
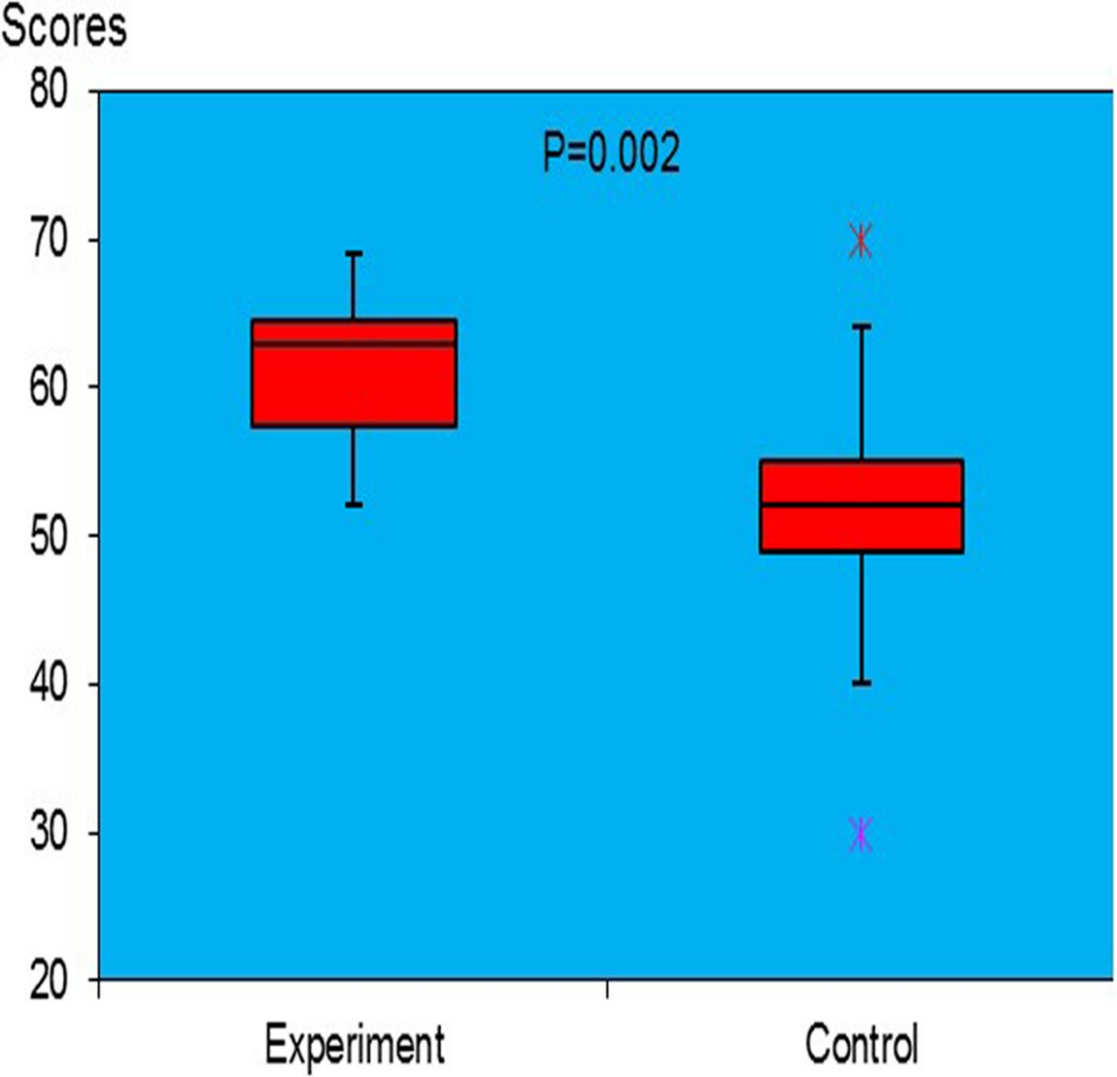
Box-and-whisker plots of the Likert questionnaire scores in both groups. The box shows the upper and lower quartile. The line in the middle is the median. The whiskers show the upper and lower limits. Crosses represent outliers.

The GRS scores of participants’ performance in the experiment group (mean score of 26.3) significantly outperformed the control group (mean score of 25.4; p = 0.046). The 10 questions in the knowledge test were divided into 3 subcategories: (i) 4 questions regarding spatial awareness (Q1, 2, 6, 10), (ii) 4 regarding operation details (Q3, 4, 5, 9) and (iii) 2 regarding sequence comprehension (Q7, 8). There was no significant difference between cohorts in the baseline test scores (for any category) before video-learning (Table 2), thus ensuring heterogeneity. Post-learning tests were repeated and are tabulated in Table 2. The 5-point Likert-scale questionnaire scores on satisfaction and acceptability (median ± IQR, 95% Bonett Price CI) in the experiment group (63±8, 59.3–66.7) was 21% significantly higher than the control group (52±7, 48.7–55.3; p = 0.002) as seen in Fig 3. After crossing over and both groups having experienced both video systems, feedback demonstrated that 80% found the multiple POV more helpful to learn the procedure compared to the single view, whereas 17% found it partially helpful and 3% found it not helpful.

**Table 2:** Overall scores and sub-scores in each category in the knowledge test before and after video learning in both groups. Score values are mean (SD) [95% CI].

Scores	Pre-video learning			Post-video learning			
	Experiment group	Control group	p-value	Experiment group	Control group	% improved	p-value
**Overall**	2.87 (1.19) [2.27–3.47]	2.73 (0.80) [2.33–3.14]	0.721	7.20 (1.08) [6.65–7.75]	5.47 (1.19) [4.87–6.07]	32	<0.001
**Spatial awareness sub-category**	1.47 (1.25) [0.84–2.10]	1.27 (0.80) [0.86–1.67]	0.605	3.07 (0.70) [2.71–3.42]	2.27 (0.96) [1.78–2.75]	35	0.023
**Operation details sub-category**	1.27 (0.80) [0.86–1.67]	0.93 (0.70) [0.58–1.29]	0.264	2.93 (0.88) [2.49–3.38]	1.67 (0.72) [1.30–2.03]	75	0.001
**Sequence comprehension sub-category**	0.47 (0.64) [0.14–0.79]	0.60 (0.63) [0.28–0.92]	0.757	1.53 (0.52) [1.27–1.79]	1.47 (0.52) [1.21–1.73]	4	0.720

## Discussion

### Uses of surgical video simulation training

Simulation is already well-established as an effective training medium in the aviation industry [24, 25]. In medicine, camera-based procedures with a single POV can be simulated in a similar fashion. Surgical video learning is a comparatively cheaper and more widely used method for trainees to gain procedural knowledge. Computer-based video instruction has been shown to be an effective indispensable tool for teaching basic surgical skills in numerous studies [26–28]. It offers several advantages over traditional teaching in the operating room, such as accommodating a large number of observers without disrupting clinical flow [29].

A variety of methods are available for capturing video of surgical procedures, but the view is frequently obscured by theatre personnel. In recent years several commercially available head mounted cameras have been used to accurately record surgeons’ view without occlusion, with early positive results [21–23]. There are also reports of Google glass being used for live broadcasting surgeries from the surgeon’s POV, which could be used for surgical training or telementoring without compromising the pace of the operation or patient safety [30, 31]. However, the downside to these head-mounted cameras is a potentially unsteady image, and a single POV. For some surgical procedures, such as joint replacement, single POV is of limited benefit, delivering a working knowledge of the operation, but not helping to develop the choreographed skillset required to perform the procedure.

### Modernizing surgical video training

In our study, we developed a novel multiple POV video system, which enabled trainees to visualize surgery from numerous viewpoints. Despite equivalent baseline test scores between the cohorts, the experiment group objectively and subjectively outperformed the control group, with an improved performance accuracy, better performance, post-learning test scores and attitude towards the learning experience. This may be attributable to a better appreciation of the technical intricacies of surgery in a 3D setting. Moreover, multiple POVs avoid loss of visualization secondary to camera occlusions. However, in terms of sub-category scores in the knowledge test post-video learning, there was no difference in sequence understanding scores between the two groups. This suggests that both video systems provided adequate information to learn the sequence of operative steps. When asked to perform part of the procedure using a sawbone simulation, the significantly lower angular deviation observed in the experiment group is an objective demonstration that the benefit of multiple POV translates into improved surgical performance, with a higher procedural accuracy. So, the null hypothesis was rejected, and with the exception of sequence comprehension and distance from optimal pin entry site, the multiple POV was found to be superior to the single POV video system.

A hip resurfacing procedure was chosen for our study for the following two reasons: (i) it is a relatively new procedure of which student or junior doctors are unlikely to have any former experience or knowledge, thus avoiding bias; and (ii) precise pin positioning in the femoral head (a key element to the success of the operation) requires visuospatial awareness and an appreciation of 3D planes from multiple angles.

Despite the advantages of the novel multiple POV, learning to operate using such a system cannot replace the clinical acumen gained from actual hands-on training. Nevertheless, our video system can act as a valuable adjunct to reinforce and enhance the learning experience. Use of this multiple POV video system may help to improve confidence and reduce anxiety before medical students and trainee surgeons move on to apprenticeship-based training in the operating room.

Whether the advantage of this multiple POV will be transferred into other surgical specialties still needs to be evaluated and is worth to be investigated in future studies.

### Limitations

Firstly, the multiple POV system appeared to stimulate more interactivity by the candidates in terms of button pressing, which may possibly encourage the participants to watch more footage of the operation than with the single POV. This could have contributed towards the higher scores in the multiple POV group. Secondly, as proposed by the participants, the multiple POV video system does not permit interaction between trainees and instructors and there was a lack of zoom function. Additionally, the Likert-scale questionnaire and the knowledge test had not been previously validated, albeit no validated alternatives exist within the medical literature. However, they were validated by four experienced orthopedic surgeons. Finally, the correlation between objective assessment of visuospatial awareness and precision of femoral head pin positioning was not included in our study, which will be conducted in the due course.

#### Future prospects

The benefit of learning surgical procedures using a multiple viewpoint over traditional single viewpoint video has been confirmed in our study. With advances in technology, the next step of our study is to immerse the trainee into a 3D reconstructed theatre setting in virtual world using the Oculus Rift VR headset (Oculus VR, California, USA). This virtual world will allow the trainee to observe an operation from any viewpoint with 3D perception.

## Conclusion

We developed a novel multiple POV video system to enhance the learning experience of orthopedic operative steps. The multiple POV provided significant objective and subjective advantages over single POV. Whereas multiple POV video cannot replace the experience of learning from actual surgery, it did enhance and reinforce the technical skills and knowledge to improve confidence, competence and performance metrics.

## Supporting information

S1 File Questionnaire on knowledge of a hip resurfacing procedure. This questionnaire evaluates the knowledge of a hip resurfacing procedure.
(DOCX)

S2 File Questionnaire to evaluate surgical video system. This questionnaire evaluates the surgical video system.
(DOCX)

S3 File Modified GRS. This questionnaire allows expert to evaluate the participants’ performance using the modified global rating scale, which was designed using a modified Delphi technique, after watching their performance recorded in the video.
(DOCX)

S4 File Dataset. This file contains the dataset of the study.
(DOCX)
