# Supplementary material for: Are multiple views superior to a single view when teaching hip surgery? A single-blinded randomized controlled trial of technical skill acquisition
Source: PLoS One. 2019 Jan 9;14(1):e0209904. doi: 10.1371/journal.pone.0209904 (PMC6326427; doi:10.1371/journal.pone.0209904)
Supplement: S1 File — This questionnaire evaluates the knowledge of a hip resurfacing procedure. (DOCX) [file pone.0209904.s001.docx]

Participant No.

Before or After:

1. **What is the position of the leg during the release of the external rotators? ( )**

*(Please note that the patient is in a lateral position i.e. lying on the left side during surgery)*

1. External rotation of the hip B. Internal rotation of the hip


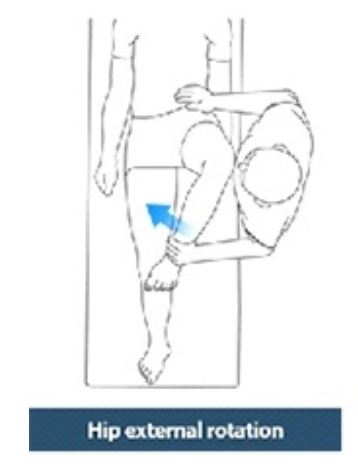

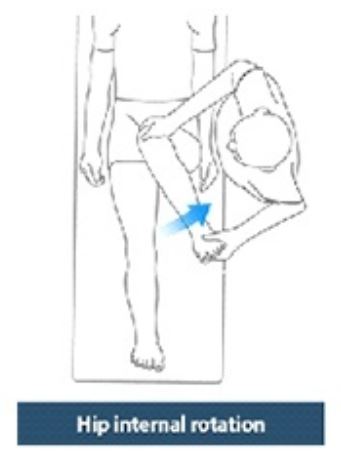


1. Adduction of the hip D. Abduction of the hip


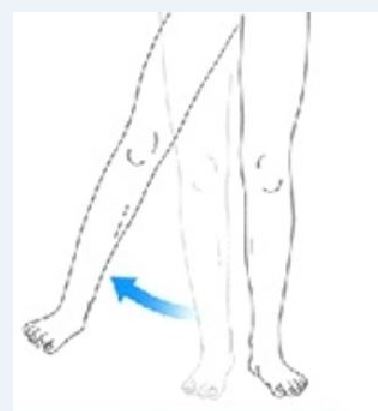

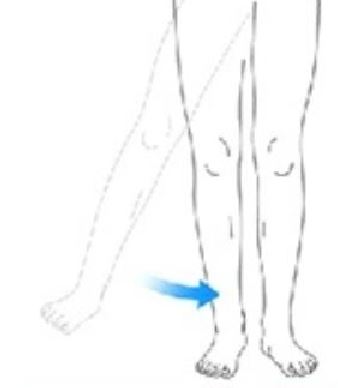


1. **What manoeuvre is used to dislocate the femoral head? ( )**

*(Please note that the patient is in a lateral position i.e. lying on the left side during surgery)*

1. More adduction and extension of the hip
2. More abduction and extension of the hip
3. More external rotation and full flexion of the hip
4. More internal rotation and full flexion of the hip
5. **What is the position of the drilled guide pin (shown below) in the femoral head? ( )**


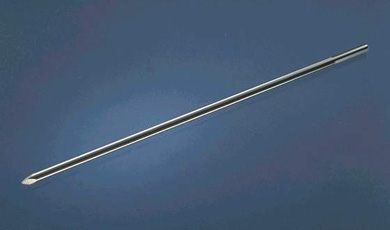


guide pin


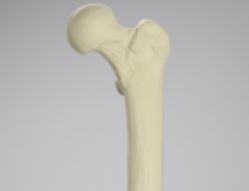


Femoral diaphysis

Femoral neck

Femoral head

1. Aiming for the centre of the femoral head
2. Aiming for the centre of the femoral neck
3. Aiming for the centre of the femoral diaphysis
4. Aiming parallel to the femoral diaphysis

4. **The following picture shows the device used to check the position of the drilled guide pin. What part does the tip of the device contact when checking pin position? ( )**

1. Equator of the femoral head
2. Osteophytes of the head
3. Femoral neck below the head
4. Intertrochanteric line


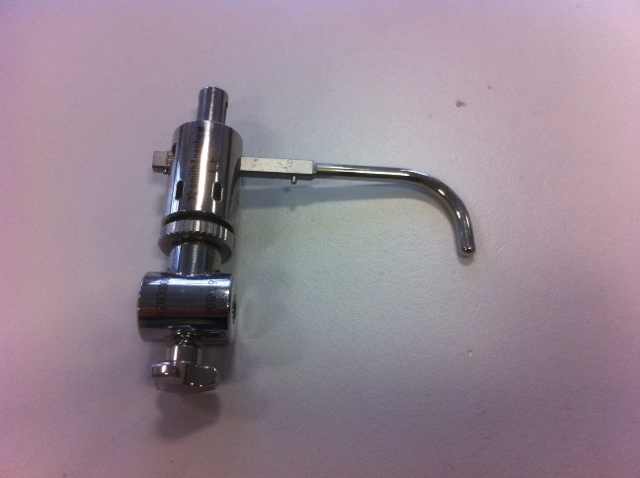


A

Tip


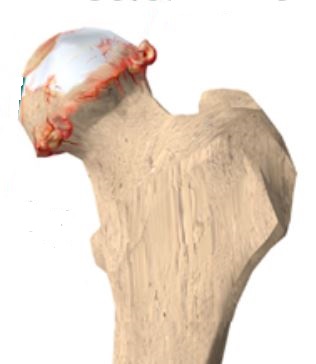


D

C

B

1. **The device shown in the picture is used for measuring the length of the femoral head. Where did the surgeon put a reference mark on the femoral head using a scalpel? ( )**


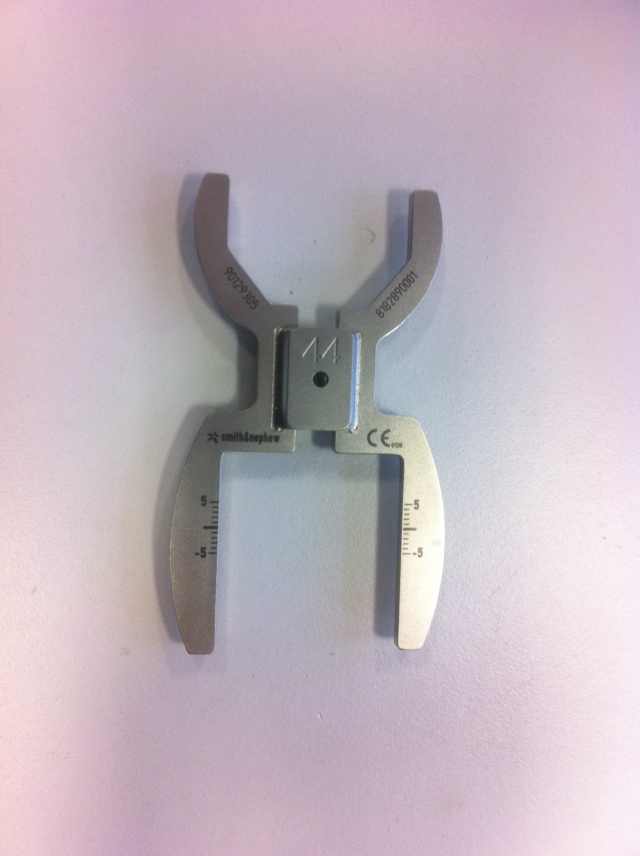


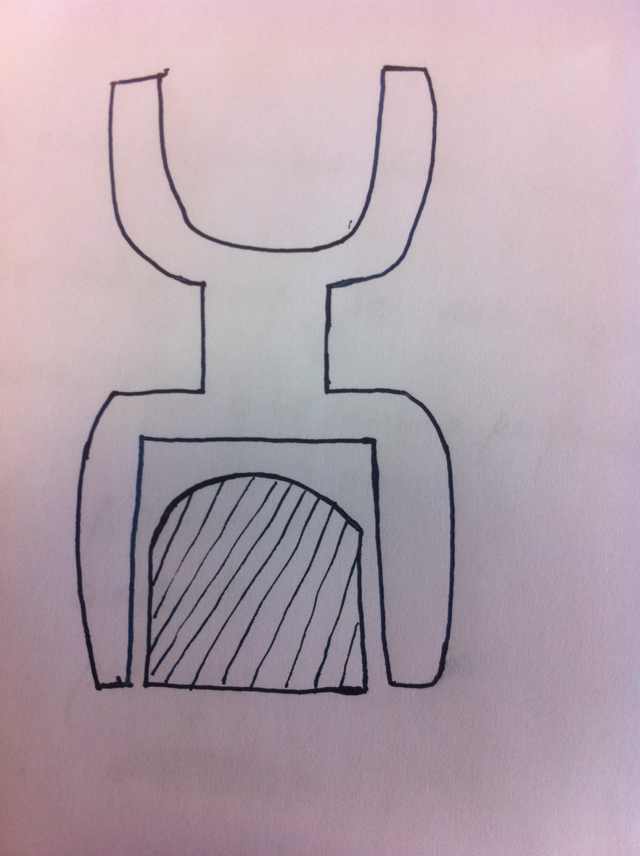


B

A

Femoral head

C

**Longitudinal section plane**

1. **What is the correct position of the reamer relative to the patient during preparation of the acetabulum (in the coronal plane)? ( )**

A.


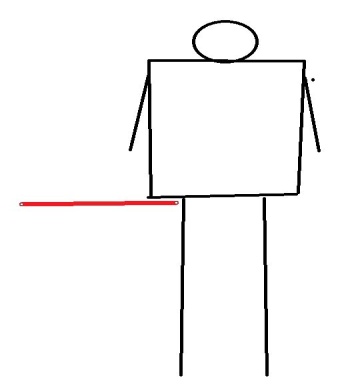


B.


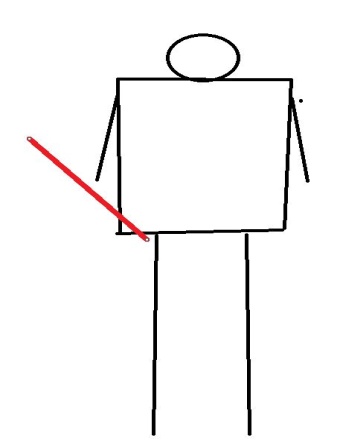


C.


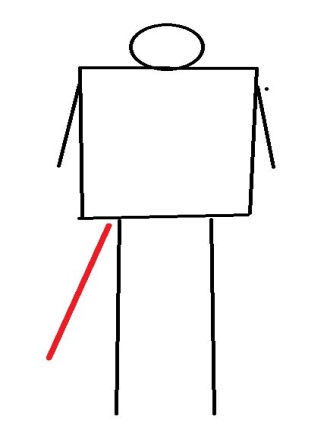


D.


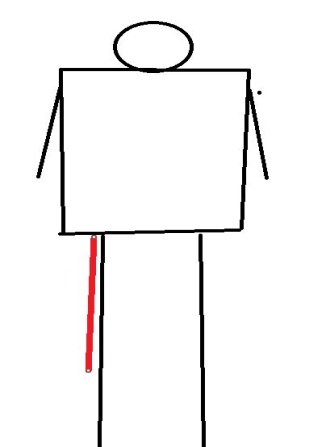


1. **Which order of steps best represents the whole procedure? ( )**

A． Dislocation of hip, preparation of femoral head, positioning of guidewire, preparation of acetabular socket

B． Dislocation of hip, positioning of guidewire, preparation of femoral head, preparation of acetabular socket

C．Dislocation of hip, preparation of acetabular socket，positioning of guidewire, preparation of femoral head

D．Dislocation of hip, preparation of acetabular socket, preparation of femoral head, positioning of guidewire

1. **Which order of steps were used to prepare the femoral head? ( )**
2. Insertion of guide-wire, measuring of the length of the head, reaming the top the head, sleeve cutting which turns the femoral head into a cylinder
3. Insertion of guide-wire, reaming the top the head, measuring of the length of the head, sleeve cutting which turns the femoral head into a cylinder
4. Insertion of guide-wire, sleeve cutting which turns the femoral head into a cylinder, measuring of the length of the head, reaming the top the head
5. Sleeve cutting which turns the femoral head into a cylinder, reaming the top the head, measuring of the length of the head
6. **The figure below shows an instrument used in the procedure. What was it used for? ( )**
7. Sleeve cutting which turns the femoral head into a cylinder
8. Reaming the acetabulum
9. Centralising the guide wire
10. Removing bone from the top of the femoral head


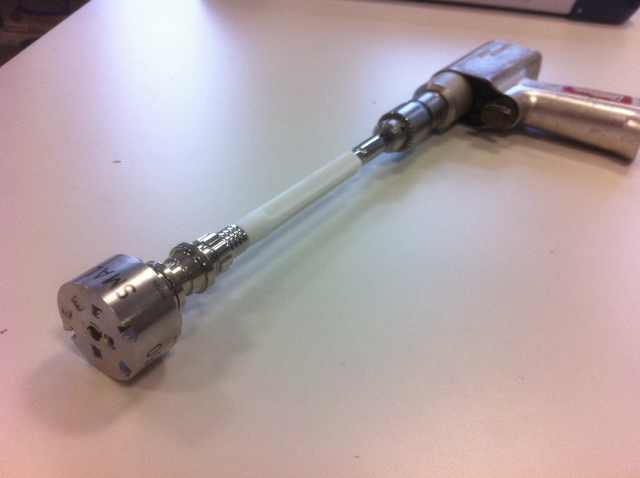


1. **What is the correct position of the square headed pin during preparation of the acetabular socket from a bird's-eye point of view? ( )**


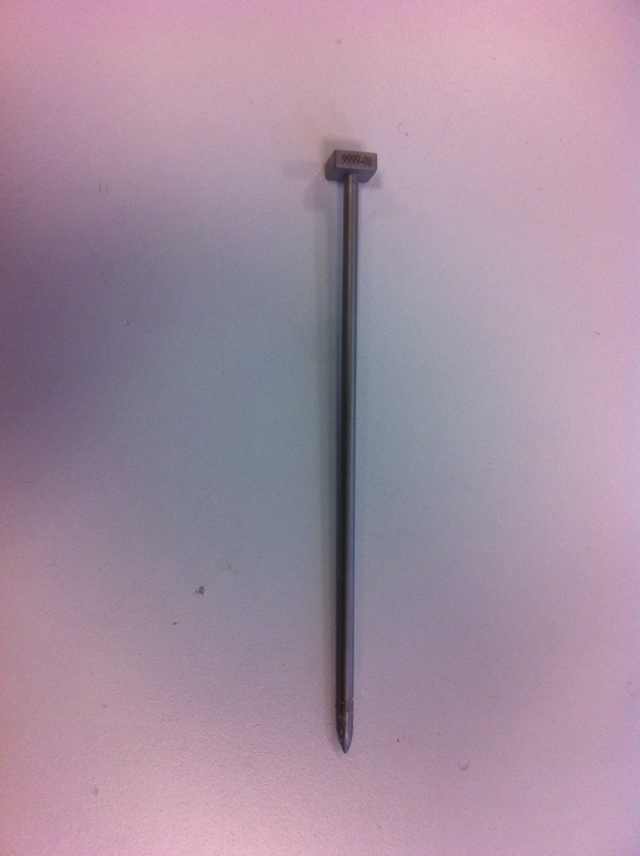


square headed pin

Surgical field

**
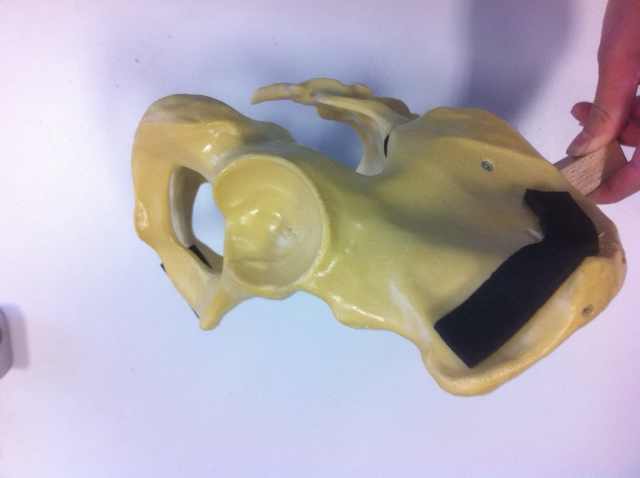
**

Posterior

Proximal

Distal

Anterior

A

C

D

B

**Demonstration of the bird's-eye view of the surgical field**
