# Supplementary material for: Are multiple views superior to a single view when teaching hip surgery? A single-blinded randomized controlled trial of technical skill acquisition
Source: PLoS One. 2019 Jan 9;14(1):e0209904. doi: 10.1371/journal.pone.0209904 (PMC6326427; doi:10.1371/journal.pone.0209904)
Supplement: S2 File — This questionnaire evaluates the surgical video system. (DOCX) [file pone.0209904.s002.docx]

**Questionnaire to evaluate surgical video system**

| What do you think of the overall quality of the images? | 1 2 3 4 5  very poor average very good |
| --- | --- |
| Were you able to visualise the surgical field clearly? | 1 2 3 4 5  very poor average very good |
| Is it easy to perceive the whole procedure? | 1 2 3 4 5  difficult average easy |
| Were you able to understand the position and direction of the drill in 3D? | 1 2 3 4 5  difficult average easy |
| Were you able to identify the anatomy in 3D? | 1 2 3 4 5  difficult average easy |
| Were you able to understand the position of the patient’s lower limb during the different steps of the operation? | 1 2 3 4 5  difficult average easy |
| Were you able to view all steps of the procedure clearly without occlusion? | 1 2 3 4 5  difficult average easy |
| Does this help you to understand the procedure before going to see it in real life? | 1 2 3 4 5  not at all partly completely |
| The surgical video(s) makes me feel immersed in the surgery, as if I was watching it in theatre. | 1 2 3 4 5  strongly disagree neutral strongly agree |
| I would recommend this to my colleagues. | 1 2 3 4 5  strongly disagree neutral strongly agree |
| I would like this type of technology to be included in my training curriculum. | 1 2 3 4 5  strongly disagree neutral strongly agree |
| I enjoyed the learning experience. | 1 2 3 4 5  strongly disagree neutral strongly agree |
| It is a useful tool for surgical training. | 1 2 3 4 5  strongly disagree neutral strongly agree |
| Watching these videos before surgery improves patient safety | 1 2 3 4 5  strongly disagree neutral strongly agree |
| Watching these videos before surgery makes me feel more confident and competent about the procedure. | 1 2 3 4 5  strongly disagree neutral strongly agree |
