# Supplementary material for: Are multiple views superior to a single view when teaching hip surgery? A single-blinded randomized controlled trial of technical skill acquisition
Source: PLoS One. 2019 Jan 9;14(1):e0209904. doi: 10.1371/journal.pone.0209904 (PMC6326427; doi:10.1371/journal.pone.0209904)
Supplement: S3 File — This questionnaire allows expert to evaluate the participants’ performance using the modified global rating scale, which was designed using a modified Delphi technique, after watching their performance recorded in the video. (DOCX) [file pone.0209904.s003.docx]

| 1. **Adequate pace and economy of movement** | 1  Taking too long and frequent unnecessary movements | 2 | 3  Efficient time /motion and not many unnecessary moves | 4 | 5  Economy of movement, maximum efficiency and ideal procedural timing |
| --- | --- | --- | --- | --- | --- |
| 1. **Instrument handling** | 1  Repeatedly makes tentative and awkward movements with instruments | 2 | 3  Competent use of instruments although occasionally appeared stiff or awkward | 4 | 5  Fluid movements with instruments without awkwardness |
| 1. **Flow of operation and forward planning** | 1  Frequently stopped operating and seemed unsure about the next move | 2 | 3  Demonstrated ability for forward planning with steady progression of operative procedure | 4 | 5  Obviously planned course of operation and with effortless flow from one operative task to the next |
| 1. **Knowledge of the procedure** | 1  Deficient knowledge and needs specific instruction at most steps | 2 | 3  Knows most principal aspects of the procedure | 4 | 5  Demonstrated familiarity with all aspects of the procedure |
| 1. **He/she placed the jig in the right direction on the femoral head（superiorly and inferiorly, anteriorly and posteriorly）** | 1  Strongly disagree | 2  Disagree | 3  neutral | 4  Agree | 5  Strongly agree |
| 1. **He/she precisely inserted pin into the femoral head** | 1  Strongly disagree | 2  Disagree | 3  neutral | 4  Agree | 5  Strongly agree |
| 1. **He/she used the devise to check the positon of the pin inserted in the femoral head properly** | 1  Strongly disagree | 2  Disagree | 3  neutral | 4  Agree | 5  Strongly agree |
| 1. **Overall performance** | 1  Very poor | 2  Poor | 3  Average | 4  Good | 5  Very good |

Modified global rating scale (GRS) for objective structured assessment of technical skills (OSATS) participants’ technical skills for the sawbone procedure
