# Supplementary material for: Are multiple views superior to a single view when teaching hip surgery? A single-blinded randomized controlled trial of technical skill acquisition
Source: PLoS One. 2019 Jan 9;14(1):e0209904. doi: 10.1371/journal.pone.0209904 (PMC6326427; doi:10.1371/journal.pone.0209904)
Supplement: S4 File — This file contains the dataset of the study. (DOCX) [file pone.0209904.s004.docx]

GRS Score Data

| **Experiment** | **Surgeon 1** | **Surgeon 2** | **Average** |  | **Control** | **Surgeon 1** | **Surgeon 2** | **Average** |
| --- | --- | --- | --- | --- | --- | --- | --- | --- |
| **1** | **28** | **27** | **27.5** |  | **1** | **24** | **26** | **25** |
| **2** | **28** | **25** | **26.5** |  | **2** | **26** | **29** | **27.5** |
| **3** | **25** | **27** | **26** |  | **3** | **23** | **25** | **24** |
| **4** | **28** | **26** | **27** |  | **4** | **25** | **25** | **25** |
| **5** | **27** | **25** | **26** |  | **5** | **28** | **26** | **27** |
| **6** | **24** | **27** | **25.5** |  | **6** | **26** | **25** | **25.5** |
| **7** | **25** | **28** | **26.5** |  | **7** | **24** | **25** | **24.5** |
| **8** | **28** | **29** | **28.5** |  | **8** | **24** | **23** | **23.5** |
| **9** | **24** | **26** | **25** |  | **9** | **26** | **27** | **26.5** |
| **10** | **28** | **29** | **28.5** |  | **10** | **25** | **24** | **24.5** |
| **11** | **27** | **25** | **26** |  | **11** | **23** | **26** | **24.5** |
| **12** | **25** | **26** | **25.5** |  | **12** | **24** | **27** | **25.5** |
| **13** | **28** | **26** | **27** |  | **13** | **28** | **25** | **26.5** |
| **14** | **23** | **25** | **24** |  | **14** | **28** | **25** | **26.5** |
| **15** | **24** | **26** | **25** |  | **15** | **24** | **25** | **24.5** |
|  |  |  | **26.3** |  |  |  |  | **25.36666667** |

Linker Questionnaire Score

| experiment group | | C1 | C2 | C3 | C4 | | C5 | | C6 | C7 | C8 | C9 | | C10 | C11 | C12 | C13 | C14 | C15 |  |
| --- | --- | --- | --- | --- | --- | --- | --- | --- | --- | --- | --- | --- | --- | --- | --- | --- | --- | --- | --- | --- |
| **1** | | 5 | 4 | 4 | 4 | | 3 | | 3 | 3 | 4 | 5 | | 4 | 4 | 4 | 5 | 3 | 3 | 58 |
| **2** | | 5 | 5 | 4 | 4 | | 4 | | 4 | 3 | 4 | 5 | | 4 | 5 | 5 | 5 | 4 | 4 | 65 |
| **3** | | 4 | 3 | 3 | 5 | | 5 | | 2 | 5 | 4 | 5 | | 4 | 4 | 3 | 4 | 3 | 3 | 57 |
| **4** | | 5 | 4 | 4 | 3 | | 3 | | 4 | 5 | 5 | 5 | | 4 | 5 | 5 | 5 | 4 | 3 | 64 |
| **5** | | 4 | 5 | 4 | 5 | | 4 | | 3 | 4 | 5 | 5 | | 5 | 4 | 4 | 4 | 4 | 4 | 64 |
| **6** | | 4 | 4 | 3 | 3 | | 3 | | 3 | 3 | 4 | 4 | | 4 | 4 | 4 | 5 | 4 | 3 | 55 |
| **7** | | 3 | 3 | 5 | 4 | | 4 | | 3 | 3 | 3 | 5 | | 3 | 3 | 3 | 5 | 4 | 5 | 56 |
| **8** | | 5 | 5 | 5 | 5 | | 4 | | 4 | 3 | 5 | 3 | | 4 | 4 | 5 | 4 | 4 | 4 | 64 |
| **9** | | 4 | 4 | 3 | 2 | | 3 | | 2 | 4 | 4 | 4 | | 4 | 4 | 4 | 4 | 3 | 3 | 52 |
| **10** | | 5 | 5 | 5 | 5 | | 3 | | 4 | 3 | 5 | 4 | | 5 | 5 | 5 | 5 | 4 | 5 | 68 |
| **11** | | 4 | 5 | 5 | 5 | | 3 | | 4 | 4 | 4 | 5 | | 4 | 4 | 5 | 4 | 3 | 4 | 63 |
| **12** | | 5 | 4 | 4 | 3 | | 4 | | 5 | 3 | 4 | 5 | | 5 | 5 | 4 | 5 | 3 | 4 | 63 |
| **13** | | 5 | 5 | 5 | 5 | | 5 | | 3 | 5 | 5 | 5 | | 4 | 5 | 3 | 5 | 5 | 4 | 69 |
| **14** | | 5 | 5 | 5 | 4 | | 4 | | 4 | 4 | 5 | 5 | | 5 | 5 | 4 | 4 | 4 | 5 | 68 |
| **15** | | 5 | 4 | 5 | 4 | | 3 | | 4 | 3 | 5 | 4 | | 4 | 5 | 5 | 4 | 4 | 3 | 62 |
| C1 | Overall quality | | | | | C9 | | Feel immersed | | | | |  |  |  |  |  |  |  |  |
| C2 | Visualize clearly | | | | | C10 | | Recommend colleague | | | | |  |  |  |  |  |  |  |  |
| C3 | Perceive procedure 3D | | | | | C11 | | Included in my curriculum | | | | |  |  |  |  |  |  |  |  |
| C4 | Understand position/direction 3D | | | | | C12 | | Enjoy experience | | | | |  |  |  |  |  |  |  |  |
| C5 | Identify anatomy 3D | | | | | C13 | | Useful tool | | | | |  |  |  |  |  |  |  |  |
| C6 | Uderstand limb position | | | | | C14 | | Improves patient safety | | | | |  |  |  |  |  |  |  |  |
| C7 | View without occlusion | | | | | C15 | | Feel confident | | | | |  |  |  |  |  |  |  |  |
| C8 | Help understand before real surgery | | | | |  | |  | | | | |  |  |  |  |  |  |  |  |

|  |  |  |  |  |  |  |  |  |  |  |  |  |  |  |  |  |
| --- | --- | --- | --- | --- | --- | --- | --- | --- | --- | --- | --- | --- | --- | --- | --- | --- |
| control group | C1 | C2 | C3 | C4 | C5 | C6 | C7 | C8 | C9 | C10 | C11 | C12 | C13 | C14 | C15 |  |
| **1** | 3 | 2 | 3 | 2 | 5 | 4 | 2 | 3 | 3 | 3 | 5 | 4 | 4 | 5 | 4 | 52 |
| **2** | 4 | 3 | 2 | 2 | 3 | 4 | 2 | 4 | 2 | 3 | 4 | 4 | 4 | 3 | 3 | 47 |
| **3** | 4 | 3 | 2 | 3 | 3 | 3 | 4 | 4 | 4 | 4 | 4 | 4 | 5 | 3 | 4 | 54 |
| **4** | 4 | 3 | 5 | 4 | 4 | 4 | 2 | 4 | 2 | 3 | 4 | 4 | 4 | 2 | 2 | 51 |
| **5** | 5 | 5 | 5 | 4 | 5 | 4 | 4 | 5 | 4 | 5 | 5 | 5 | 4 | 5 | 5 | 70 |
| **6** | 4 | 3 | 3 | 3 | 2 | 3 | 3 | 4 | 4 | 5 | 5 | 5 | 5 | 4 | 5 | 58 |
| **7** | 4 | 3 | 4 | 3 | 4 | 4 | 2 | 4 | 4 | 4 | 4 | 4 | 3 | 4 | 3 | 54 |
| **8** | 2 | 2 | 1 | 1 | 1 | 2 | 1 | 2 | 2 | 2 | 4 | 2 | 2 | 3 | 3 | 30 |
| **9** | 4 | 3 | 2 | 3 | 3 | 4 | 3 | 4 | 4 | 4 | 5 | 5 | 4 | 4 | 4 | 56 |
| **10** | 3 | 2 | 2 | 3 | 3 | 4 | 3 | 4 | 3 | 3 | 4 | 4 | 4 | 4 | 3 | 49 |
| **11** | 5 | 4 | 2 | 2 | 2 | 3 | 2 | 3 | 3 | 3 | 4 | 4 | 4 | 4 | 4 | 49 |
| **12** | 5 | 2 | 1 | 2 | 2 | 2 | 3 | 4 | 5 | 4 | 5 | 4 | 4 | 5 | 4 | 52 |
| **13** | 4 | 2 | 2 | 2 | 2 | 3 | 3 | 4 | 4 | 3 | 2 | \| 5 \| \| --- \| | 4 | 3 | 4 | 47 |
| **14** | 5 | 4 | 4 | 4 | 5 | 5 | 4 | 5 | 5 | 5 | 4 | 4 | 5 | 5 | 5 | 69 |
| **15** | 4 | 3 | 3 | 4 | 3 | 4 | 2 | 3 | 4 | 4 | 4 | 4 | 4 | 2 | 2 | 50 |

| C1 | Overall quality | C9 | Feel immersed |
| --- | --- | --- | --- |
| C2 | Visualize clearly | C10 | Recommend colleague |
| C3 | Perceive procedure 3D | C11 | Included in my curriculum |
| C4 | Understand position/direction 3D | C12 | Enjoy experience |
| C5 | Identify anatomy 3D | C13 | Useful tool |
| C6 | Understand limb position | C14 | Improves patient safety |
| C7 | View without occlusion | C15 | Feel confident |
| C8 | Help understand before real surgery |  |  |

Objective data

| **Experiment** | **distance of entry point** | **angle deviation** |  | **Control** | **distance of entry point** | **angle deviation** |
| --- | --- | --- | --- | --- | --- | --- |
| **1** | **4.13** | **5.5** |  | **1** | **8.23** | **7.95** |
| **2** | **4.33** | **4.45** |  | **2** | **13.58** | **15.88** |
| **3** | **2.63** | **3.94** |  | **3** | **7.73** | **8.63** |
| **4** | **8.68** | **8.1** |  | **4** | **8.32** | **6.83** |
| **5** | **4.03** | **2.98** |  | **5** | **3.78** | **8.09** |
| **6** | **5.01** | **3.35** |  | **6** | **4.07** | **4.82** |
| **7** | **5.35** | **10.08** |  | **7** | **5.87** | **7.74** |
| **8** | **5.25** | **6.45** |  | **8** | **7.19** | **7.18** |
| **9** | **11.34** | **10.6** |  | **9** | **7.74** | **7.09** |
| **10** | **5.22** | **4.82** |  | **10** | **5.19** | **8.78** |
| **11** | **4.6** | **5.33** |  | **11** | **6.18** | **13** |
| **12** | **0.89** | **1.86** |  | **12** | **9.84** | **9.95** |
| **13** | **8.63** | **13.71** |  | **13** | **5.33** | **14.31** |
| **14** | **6.63** | **3.99** |  | **14** | **2.34** | **7.09** |
| **15** | **5.87** | **10.68** |  | **15** | **6.05** | **7.6** |

Quiz (before)

|  | spatial awareness | operation details | Sequence  understanding | | spatial awareness | operation details | sequence understanding |
| --- | --- | --- | --- | --- | --- | --- | --- |
| experiment（before） | sub-score 4 | sub-score 5 | sub-score 6 | control（before） | sub-score 4 | sub-score 5 | sub-score 6 |
| 1 | 2 | 2 | 0 | 1 | 1 | 1 | 0 |
| 2 | 0 | 1 | 0 | 2 | 2 | 2 | 0 |
| 3 | 1 | 1 | 1 | 3 | 1 | 1 | 1 |
| 4 | 2 | 1 | 0 | 4 | 0 | 0 | 1 |
| 5 | 0 | 1 | 2 | 5 | 3 | 0 | 1 |
| 6 | 2 | 2 | 1 | 6 | 2 | 1 | 0 |
| 7 | 1 | 1 | 0 | 7 | 2 | 0 | 1 |
| 8 | 1 | 3 | 0 | 8 | 1 | 1 | 0 |
| 9 | 0 | 1 | 1 | 9 | 1 | 2 | 1 |
| 10 | 2 | 1 | 1 | 10 | 1 | 2 | 0 |
| 11 | 0 | 2 | 0 | 11 | 1 | 1 | 1 |
| 12 | 4 | 0 | 0 | 12 | 2 | 0 | 0 |
| 13 | 3 | 2 | 0 | 13 | 1 | 1 | 1 |
| 14 | 3 | 0 | 1 | 14 | 1 | 1 | 0 |
| 15 | 1 | 1 | 0 | 15 | 0 | 1 | 2 |
|  | 1.466666667 | 1.266666667 | 0.466666667 |  | 1.266666667 | 0.933333333 | 0.6 |

Quiz (After)

|  | spatial awareness | operation details | Sequence  understanding | | spatial awareness | operation details | sequence understanding |
| --- | --- | --- | --- | --- | --- | --- | --- |
| experiment（after） | sub-score 1 | sub-score 2 | sub-score 3 | control（after） | sub-score 1 | sub-score 2 | sub-score 3 |
| 1 | 3 | 3 | 2 | 1 | 1 | 1 | 1 |
| 2 | 3 | 2 | 2 | 2 | 3 | 3 | 2 |
| 3 | 3 | 2 | 2 | 3 | 3 | 1 | 2 |
| 4 | 3 | 4 | 1 | 4 | 3 | 1 | 2 |
| 5 | 4 | 3 | 1 | 5 | 3 | 1 | 2 |
| 6 | 3 | 4 | 2 | 6 | 1 | 2 | 2 |
| 7 | 4 | 2 | 1 | 7 | 2 | 3 | 1 |
| 8 | 4 | 3 | 2 | 8 | 1 | 2 | 2 |
| 9 | 3 | 2 | 1 | 9 | 2 | 1 | 1 |
| 10 | 2 | 4 | 1 | 10 | 2 | 2 | 1 |
| 11 | 3 | 2 | 1 | 11 | 2 | 2 | 2 |
| 12 | 2 | 3 | 2 | 12 | 3 | 1 | 1 |
| 13 | 3 | 4 | 1 | 13 | 3 | 1 | 1 |
| 14 | 4 | 2 | 2 | 14 | 4 | 2 | 1 |
| 15 | 2 | 4 | 2 | 15 | 1 | 2 | 1 |
|  | 3.066666667 | 2.933333333 | 1.5333333 |  | 2.26666667 | 1.666666667 | 1.466667 |

Videos access

The videos are available on the Dryad data repository:

<https://doi.org/10.5061/dryad.qr60ps0>

This repository contains the videos used in the article "Are multiple views superior to a single view when teaching hip surgery? A single-blinded randomized controlled trial of technical skill acquisition".

Two sequences are present, the dry bone sequence and the surgery sequence. The dry bone sequence’s filenames begin with “drybone_cam” and the surgery sequence’s filenames begin with “surgery_cam”.

There are 7 videos for the dry bone sequence and 8 videos for the surgery sequence. Each video represents a different point of view of the sequence.

The videos have a size of 1440x810 pixels. The codec for the video is MP4 and there are 25 frames per second.

The audio codec is AAC at 48 kHz.
